# Self-Regulation and Psychological Well-Being in Early Adolescence: A Two-Wave Longitudinal Study

**DOI:** 10.3390/bs10030067

**Published:** 2020-03-10

**Authors:** Tatiana Fomina, Angelika Burmistrova-Savenkova, Varvara Morosanova

**Affiliations:** Psychological Institute of the Russian Academy of Education, Mokhovaya st. 9, bld.4, Moscow 125009, Russia; cygnet@inbox.ru (A.B.-S.); morosanova@mail.ru (V.M.)

**Keywords:** psychological well-being, self-regulation, cross-lagged analysis

## Abstract

This paper addresses the question of whether self-regulation capacities are a significant psychological resource of schoolchildren’s psychological well-being. The study contributes to the search of significant predictors of the students’ psychological well-being. Moscow secondary schools pupils (N = 239) participated in a two-wave longitudinal study, the procedure being made in the 4th grade and repeated in the 5th grade, six months after the first measurement. The results are presented describing the dynamics of manifestations of the psychological well-being and the conscious self-regulation of the schoolchildren during their transition from the primary to the middle school. Using the cross-lagged panel analysis allowed concluding that the level of conscious self-regulation of the learning activity of the 4th graders significantly predicts their psychological well-being not only in the 4th grade, but also in the 5th grade. The study revealed the specific regulatory predictors characteristic of different manifestations of the schoolchildren’ psychological well-being. The obtained results highlight the significance of research on the conscious self-regulation of learning activities as a resource for pupils’ psychological well-being, which is predictive for its maturation in the subsequent ages.

## 1. Introduction

The study of the students’ psychological well-being is currently an urgent theoretical and practical task. The level of the schoolchildren’s psychological well-being is considered as an indicator of the social welfare and a measure of the educational system effectiveness [1,2,3,4]. We consider psychological well-being (PWB) in the frame of the phenomenological approach based on the well-being manifestations in everyday life. It is not limited to relationships with oneself but also includes social involvement, motivation, and positive interactions with other people [5]. Students’ well-being is closely associated with a positive school environment, which involves the social and academic adaptation of students. Many studies have shown that adolescents are mostly satisfied with their lives as a whole and that satisfaction in the school environment is essential for the PWB of 10 to 12-year-old adolescents [6,7]. Many researchers point out significant changes in the PWB of schoolchildren during their transition from the primary to the middle school [8,9]. Being positively associated with academic success, positive peer relationships, and lower manifestations of stress in the academic environment, PWB is a prerequisite for adolescents’ mental and physical health [10,11,12]. Adolescents with high levels of well-being are more resilient [13], present lower delinquency behaviors and aggression, lower depressive and anxiety symptoms, higher self-esteem, self-efficacy, and adaptation [14,15,16]. It is supposed that during the school period, the foundations are laid for the positive functioning in adulthood [4,17]. That is why many researchers highlight that the promotion of the students’ well-being needs to be established as an educational priority [18,19,20].

Research demonstrated that self-regulation in various forms is associated with students’ PWB [21,22,23,24]. Self-regulation is a broad term, denoting any regulation of the self by the self; thus, whenever by use of some psychological capacity, some psychological process—be it behavioral, motivational, or attentional—is brought to the desired state, this is an instance of self-regulation [25]. Self-regulation learning strategies, as well as goal orientation and intrinsic value, positively predicted the students’ PWB. Based on research results, the teaching of self-regulated learning strategies for students is recommended as a promotive strategy in mental health [26,27]. For example, the results of the very few longitudinal studies show that developed self-regulation skills in adolescents predict a higher level of positive affect and well-being as a whole [28,29]. In a dynamic context, a positive impact on the well-being in subsequent periods is also found for such characteristics as school engagement [30,31], achievement goal orientations [27], character strengths [32], and social connectedness [33]. The longitudinal design of the studies allows for identifying the reciprocal relationship between the studied characteristics. There is every reason to believe that the relationship between self-regulation and PWB may be reciprocal. Thus, it has been shown that social exclusion and self-regulation reciprocally affect one another over time. Social exclusion undermines children’s development of self-regulation, whereas poor self-regulation increases the likelihood of exclusion [34]. However, the research of the students’ PWB is still insufficient since both certain age groups and potential prognostic predictors of PWB remain uncovered. Therefore, the study of the PWB dynamics and its regulatory resources is an urgent research task that can help to identify the psychological mechanisms for maintaining PWB in different periods of schooling. A comprehensive study would answer the questions about the long-term impact of individual characteristics on the students’ well-being.

The above circumstances may affect the students’ PWB and, at the same time, make high demands on their conscious self-regulation—their ability to effectively, reliably, and flexibly act in the new conditions, retaining activity goals, building action programs, and evaluating results of the learning activities. We consider conscious self-regulation (SR) as an integrative cognitive–intrapersonal construct. It represents a cognitive system of information processing, including goal planning, the modeling of significant conditions, programming of actions, and results evaluating. At the same time, it is represented by the peculiarity of instrumental personality–regulatory properties: flexibility, independence, reliability, responsibility, etc. This structure of conscious SR emphasizes the meta-nature of this psychological means of mobilizing and integrating both cognitive and personal resources to solve educational tasks [35]. Within the framework of our research domain, we presented several studies demonstrating significant relationships between the schoolchildren’s conscious SR and their well-being indicators [36,37].

The present study delivers results obtained from the longitudinal data demonstrating the dynamics of PWB and the impact of the conscious SR development on the PWB of the pupils during their transition from the 4th to the 5th grade. In Russian schools, the 4th grade completes the primary stage of education, after which all the children go to the middle stage of secondary school. In the 5th grade, the educational process conditions change significantly: the number of subjects in the curriculum increases; the teaching stuff expands; the new classmates appear as after the primary stage, some parents decide to change the school for different reasons. As a result, the load on the schoolchildren’s adaptive capacities and regulatory abilities drastically increases. It is during this period that different researchers record the changes in the level and quality of PWB in the samples of pupils from various countries [38].

Thus, in the present study, it was supposed to answer a series of questions. First, what is the dynamics of the conscious SR and PWB of the schoolchildren during their transition from the 4th to the 5th grade? Second, how are specific parameters of the conscious SR related to the pupils’ PWB at this stage of education? Finally, is it possible to consider conscious SR to be a long-time predictor of the different PWB aspects when moving from primary to middle school?

## 2. Materials and Methods

### 2.1. Participants

The sample consisted of secondary school students in Moscow and the Moscow region. At T1, 298 people were examined—4th grade pupils, 51% males, aged from 10 to 12 years (M = 10.31, SD = 0.48). At T2, the same groups were examined six months later, when they moved from the primary to the middle school, and there were 293 people—5th grade students, 49.8% males, aged from 10 to 12 years (M = 10.62, SD = 0.50). All in all, 239 people (48% males) completed the survey at T1 and T2.

Parental and school consent was obtained for all participants. Analyses were carried out on the depersonalized data. The study was conducted in accordance with the Helsinki Declaration. Ethical agreement and consent for access to school were provided by the Ethics Committee of the Psychological Institute of the Russian Academy of Education (approval number 2017/1-128).

### 2.2. Self-Regulation

Morosanova’s Self-Regulation Profile Questionnaire—Junior [39] consists of 7 self-assessment scales: Planning the goals (e.g., “I know what grades I want to get at the end of the year”), Modeling significant conditions (e.g., “Prior to start solving the task, I always carefully examine its introductory conditions”), Programming Activity (e.g., “I have no difficulty in drawing up a plan of presentation”), Evaluating Results (e.g., “I rarely notice my mistakes”), Flexibility (e.g., “I’m back to studies quickly after the holidays”), Independence (e.g., “I usually do my homework by myself”), and Responsibility (e.g., “I seek to making additional tasks”). Each item is scored on a 6-point scale with responses from 1 = “not at all like me” to 6 = “very much like me”. The pupils are to choose to what extent the described behavior is characteristic of them. The general SR level is estimated by summing up the scores on seven scales. The incentive material is presented in the form of descriptions of typical situations that are associated with the organization of the learning activities and pupils’ behavior relative to the training implementation and are accessible for primary school age. The coefficients of internal consistency of the items for each scale range from 0.62 to 0.79, indicating an overall reasonable homogeneity of the items in each scale.

### 2.3. Psychological Well-Being

PWB was accessed using the slightly modified Russian adaptation of the 25-item Well-Being Manifestation Measure Scale developed by Masse [5]. The questionnaire was previously validated on the sample of the 4th grade pupils in the Russian secondary schools [40]. The participants were asked to evaluate to which extent they experienced the described states over the past month on a scale ranging from 1 (“never”) to 5 (“almost always”). This questionnaire contains 25 statements divided into 6 subscales: 4 statements for “Control of Self and Events” (e.g., “I was able to face difficult situations in a positive way”); 5 statements for “Happiness” (e.g., “I found life exciting and I wanted to enjoy every moment of it”), 4 statements for “Social Involvement” (e.g., “I felt like having fun, doing sports, and participating in all my favorite activities and past-times”); 4 statements for “Self-Esteem” (e.g., “I had self-confidence”); 4 statements for “Mental Balance” (e.g., “My life was well-balanced between my family, personal, and school activities”); 4 statements for “Sociability” (e.g., “I got along well with everyone around me”). The cumulative scale “Psychological Well-Being” is calculated by summing up the scores on all the scales. The internal reliability coefficients for the present study were 0.72–0.78.

### 2.4. Statistical Analysis

At the first stage, the descriptive statistics concerning the variables under study were analyzed with an assessment of the significance of their changes from grade 4 to grade 5 and the calculation of the effect size of the differences obtained. In the second stage, the lagged regression models were analyzed. Cross-lagged panel analysis was selected as the most appropriate instrument to solve the research task. Based upon an assumption that one event consistently takes precedence of the occurrence of another event, this analytic technique allows the researcher to differentiate and disentangle the relative plausibility and strength of competing for causal interpretations between two variables measured on two different occasions.

## 3. Results

### 3.1. Descriptive Statistics

Table 1 presents descriptive statistics (average, standard deviations) for indicators of conscious SR and PWB among the pupils, which were measured in the 4th and 5th grades. In addition, using the variance analysis, we estimated the significance of changes in all parameters as well as the effect size of the differences obtained. No significant gender differences in the studied parameters were found.

According to the data obtained, certain changes are observed between the indicators measured in the 4th and 5th grades. There is an increase in all indicators of the pupils’ PWB (except for “Sociability”), as well as in mostly all regulatory indicators (except for “Modeling significant conditions”). These changes are significant for the indicators of planning, programming, flexibility, responsibility, and the general level of conscious SR. The largest effect size of the resulting differences is fixed for “Control of self and events” scale. This is primarily due to the increased autonomy of the schoolchildren, as well as responsibility for organizing their learning activities. The magnitude of the effect size of the detected differences according to the Cohen scale refers to “weak” and “medium”. The largest effect size is observed for the regulatory–personal characteristic “flexibility”, which suggests that in the 5th grade, schoolchildren are faced with a need to update their ability to respond quickly to the changing learning activities, flexibly regulating their behavior.

### 3.2. Cross-Lagged Correlation

**Figure 1:**
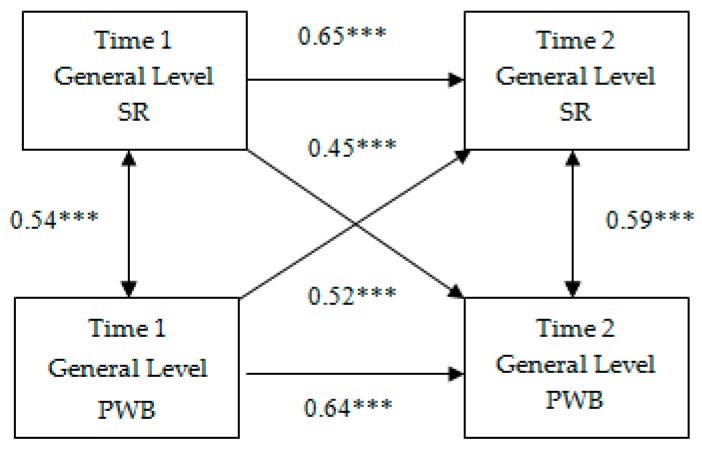
Cross-lagged relationships between self-regulation and psychological well-being. *** = *p* < 0.001; SR = self-regulation; PWB = psychological well-being.

At the next stage, we used correlation analysis to assess the stability and variability of the interrelationships of the conscious SR general level and the general PWB level of the pupils during their transition from primary to middle school. As shown in Figure 1, there are six possible relationships among T1 and T2 variables within the cross-lagged analytic model (see [41]). The two diagonal lines represent path coefficients between T1 Self-regulation and T2 Well-being factors and between T1 Well-being and T2 Self-regulation factors, respectively. These are “cross-lagged relationships” (lag refers to a time interval between two measurements). The two horizontal lines represent the path coefficients between the same variables measured on two occasions, e.g., between T1 SR and T2 PWB. These are “auto-lagged relationships”. The two double-arrowed lines represent the correlation coefficients between variables measured on the same occasion (T1 panel and T2 panel).

The obtained correlation coefficients allow drawing the following conclusions: first, the significant interrelationship between the pupils’ SR and PWB is reproduced within the age cuts; second, the significant autoregressive correlation coefficients indicate the relative stability of the studied constructs; and third, the cross-lagged relationships between SR and PWB are significant. Besides, a reciprocal relationship is observed between the conscious SR and PWB.

### 3.3. A lagged Regression Analysis

Next, a regression analysis was conducted to answer the question: Can self-regulation serve as a significant long-term predictor of the schoolchildren’s well-being? Besides, which regulatory features may be more important for different aspects of pupils’ PWB? The independent variables were seven self-regulation indicators measured in the 4th grade. The dependent variables were indicators of the pupils’ PWB in the 5th grade. Thus, seven regression models were estimated for all manifestations of PWB. They are presented in Table 2.

The obtained results suggest that for different aspects of the pupils’ PWB, there exist specific regulatory predictors. Attention is drawn to the fact that the regulatory process “programming” turned to be a predictor of the most indicators of pupils’ PWB. “Social involvement” is the only indicator for which no significant regulatory predictors were found. For the general level of PWB, the prognostic factors were indicators of planning, programming, and result evaluating). Thus, the data obtained suggest that the development of conscious SR and its particular components is, to a certain extent, a significant prognostic resource for both the general level of PWB and its different aspects.

## 4. Discussion

The results obtained in the present research clarify certain aspects of the relationship between the conscious SR of early adolescents and their PWB. The longitudinal design of the study made it possible to demonstrate that the majority of schoolchildren in the 5th grade undergo significant changes in the direction of increasing most indicators of both conscious SR and PWB compared to the period of their study in the 4th grade. It is, in our opinion, connected with the fact that during their transition from the primary to the middle school, pupils receive significant social support from parents and teachers due to adaptation to the new period of schooling. At the same time, finding themselves in the changed situation, they acquire unique experience in the self-organization of their activities and social interaction, becoming more independent and autonomous in solving educational tasks. This critical experience becomes the basis for a positive self-attitude. The increase in the level of regulatory indicators takes place primarily due to the age-related specifics and the social situation of development in which the maturation of the conscious SR becomes one of the new growths of adolescence [42,43]. Transition to the middle stage of the secondary school may represent an opportunity for developing interventions aimed at improving both pupil psychological functioning and attainment [44].

Many researchers highlight the need to study psychological mechanisms originating from childhood and adolescence that are the basis for the PWB of adults [45,46]. As the most promising in this regard, they consider personality traits, self-control, and self-regulation. However, the longitudinal studies on this subject are few. Many studies demonstrate that self-regulation prevents the emergence of negative scenarios in the development of adolescents, in particular, manifestations of depression and delinquent behavior [47], and it also reduces the likelihood of a problem and risky behavior [48]. Another study is focused on prognostic relationships of adolescents’ life satisfaction and school engagement, which is considered as a meta-construct, including behavioral, cognitive, and emotional components [49]. In this case, the cognitive side of the school engagement is associated with the self-regulation of the learning activity. Studies have shown that during the transition from primary to middle school, it is the cognitive aspect of engagement measured in primary school age that significantly predicts the adolescents’ life satisfaction in subsequent periods [30].

Our results confirm the already obtained data on the role of SR in the dynamics of the schoolchildren’s well-being and contribute to the further development of this research problem. Based on the longitudinal data, we demonstrated the significant role of the conscious SR development in the prediction of the young adolescents’ PWB. In our previous studies, we obtained similar data on the positive impact of the pupils’ conscious SR in the 4th to 5th grades on their subjective well-being [50]. The present study has shown that particular regulatory predictors have their specifics concerning different PWB aspects, and the most significant role in these relationships belongs to the regulatory processes of planning, programming, and results evaluating.

The value of the obtained data consists of the identification of specific regulatory characteristics that are significant for predicting various manifestations of adolescents’ PWB. However, some regulatory features turned out to be not significant statistically. For example, for such a manifestation of PWB as “social involvement”, none of the regulatory characteristics was found to be a significant predictor. We assume that this may be because the social activity of adolescents is determined to a greater extent by the other factors. For example, this may be related to personality traits and a need for communication [51]. The regulatory processes of planning and modeling were also not significant for PWB manifestations. Presumably, these processes in adolescents are in the stage of active development. Indeed, adolescence is a crucial period for the development of conscious self-regulation [52]. It is possible that we will find significant effects of these regulatory characteristics at subsequent ages when they are sufficiently formed. Regulatory properties (independence and responsibility) did not reach the level of statistical significance in the prognosis of adolescents’ PWB. In this case, it can be assumed that PWB manifestations are weakly related to the adolescent’s ability to autonomously and independently regulate their activities. At this age, children regulate their activity relating to adults or peers [53]. PWB also does not depend on how responsible adolescents are in performing their duties, and the degree of responsibility development is not critical for the PWB. The researchers consider self-regulation as a global process in early adolescence that contributes to positive development [54]. Therefore, further research in this area seems promising. Our study results open the perspective for investigating the specifics of these relationships in the students of older age groups.

## 5. Limitations and Directions for Future Research

Although the findings are promising, our study also has several limitations. First, our longitudinal study is limited by two time slices. In future research, it is necessary to evaluate the identified effects for the more extended time periods. Secondly, we used the technique allowing for the assessment of manifestations of the schoolchildren PWB, which imposes limitations on the conclusions regarding the impact of the school environment on it. Thirdly, our study did not take into account the external factors relevant both for the pupils’ SR development and for their PWB (e.g., school climate, peer group relationships, academic achievement). In the context of future research, it seems necessary to study individual trajectories in the development of SR and PWB, as well as the role of the conscious SR as a mediator in the relationship between personality factors and PWB of students in different ages.

## 6. Conclusions

Despite these limitations, the present study makes a valuable contribution to the literature on self-regulation and well-being. The data are obtained showing the positive dynamics of the pupils’ PWB during their transition from the primary to the middle school. The study revealed the significant influence of the conscious SR development on various manifestations of the schoolchildren’s PWB. In early adolescence, conscious SR of the learning activity can play the role of PWB resources at the next stages of education. In this connection, the most relevant regulatory features to be developed in the primary school age are planning goals, programming actions, results evaluating, and also flexibility as an ability to correct the activity in the changing conditions.

## Figures and Tables

**Table 1:** Descriptive statistics. PWB: psychological well-being, SR: self-regulation.

Variables	4th Grade		5th Grade		*ANOVA*	*Cohen’s d*
	*M*	*SD*	*M*	*SD*		
Control of self and events	13.52	3.40	14.52	3.35	−4.09 ***	−0.30
Happiness	18.95	4.24	19.54	4.05	−2.06 *	−0.14
Social involvement	14.73	3.18	15.25	3.21	−2.14 *	−0.16
Self-esteem	13.59	3.33	14.15	3.49	−2.56 *	−0.16
Mental balance	14.45	3.35	15.00	3.11	−2.43 *	−0.17
Sociability	15.33	3.20	15.45	3.28	−0.31	−0.04
PWB general level	90.59	16.60	93.52	17.71	−2.98 **	−0.17
Planning of goals	4.35	1.26	4.56	1.21	−2.44 *	−0.17
Modeling	4.55	1.32	4.15	1.48	3.79 ***	0.28
Programming	4.20	1.26	4.34	1.26	−1.58	−0.11
Results evaluation	3.89	1.31	3.99	1.36	−0.96	−0.07
Flexibility	3.83	1.54	4.20	1.43	−3.09 **	−0.25
Independence	4.35	1.31	4.46	1.33	−1.21	−0.08
Responsibility	4.21	1.25	4.47	1.21	−3.44 ***	−0.21
SR general level	29.41	5.97	30.17	5.70	−2.48 *	−0.13

* = *p* < 0.05, ** = *p* < 0.01, *** = *p* < 0.001.

**Table 2:** Summary of a lagged regression analysis for the variables predicting psychological well-being (N = 235).

Control Variables	Model 1 Control of Self and Events			Model 2 Happiness			Model 3 Social Involvement		
	*B*	*SEB*	*t*	*B*	*SEB*	*t*	*B*	*SEB*	*T*
Planning	0.360	0.202	1.787	0.260	0.250	0.081	0.367	0.198	1.853
Modeling	0.221	0.174	1.269	0.000	0.215	0.000	0.014	0.171	0.084
Programming	0.476	0.197	2.412 *	0.417	0.244	0.130	0.375	0.194	1.936
Result Evaluation	0.298	0.179	1.658	0.455	0.222	0.148 *	0.129	0.176	0.731
Flexibility	0.141	0.148	0.956	0.037	0.183	0.014	0.079	0.145	0.547
Independence	0.055	0.163	0.335	0.153	0.202	0.050	0.211	0.160	1.316
Responsibility	0.309	0.198	1.565	0.282	0.245	0.088	0.080	0.194	0.411
R^2^	0.173			0.128			0.124		
p	<0.0001			<0.0001			<0.0001		
	**Model 4** **Self-Esteem**			**Model 5** **Mental Balance**			**Model 6** **Sociability**		
	*B*	*SEB*	*t*	*B*	*SEB*	*t*	*B*	*SEB*	*T*
Planning	0.389	0.204	1.903	0.375	0.184	2.037	0.111	0.197	0.564
Modeling	0.100	0.176	0.567	0.206	0.159	1.294	0.072	0.170	0.426
Programming	0.596	0.200	2.983 **	0.593	0.180	3.293 **	0.620	0.193	3.212 **
Result Evaluation	0.427	0.182	2.347 *	0.051	0.164	0.311	0.210	0.176	1.195
Flexibility	0.035	0.150	0.235	0.108	0.135	0.801	0.198	0.145	1.366
Independence	0.018	0.165	0.109	–0.227	0.149	–1.527	0.021	0.159	0.131
Responsibility	0.320	0.200	1.599	0.183	0.180	1.014	0.192	0.193	0.995
R^2^	0.209			0.195			0.168		
p	<0.0001			<0.0001			<0.0001		
	**Model 7 PWB General Level**								
	*B*			*SEB*			*T*		
Planning	2.300			1.002			2.295 *		
Modeling	0.031			0.864			0.468		
Programming	3.388			0.982			3.449 ***		
Result Evaluation	1.878			0.893			2.102 *		
Flexibility	0.490			0.738			0.664		
Independence	0.167			0.813			0.205		
Responsibility	1.297			0.986			1.316		
R^2^	0.250								
p	<0.0001								

* = *p* < 0.05, ** = *p* < 0.01, *** = *p* < 0.001.
